# The Effects of Old Age on Hepatic Stellate Cells

**DOI:** 10.1155/2011/439835

**Published:** 2011-05-18

**Authors:** Alessandra Warren, Victoria C. Cogger, Robin Fraser, Laurie D. DeLeve, Robert S. McCuskey, David G. Le Couteur

**Affiliations:** ^1^Centre for Education and Research on Ageing, Concord Hospital and the ANZAC Research Institute, The University of Sydney, Sydney NSW 2139, Australia; ^2^Department of Pathology, University of Otago and Christchurch Hospital, Christchurch 8140, New Zealand; ^3^Division of Gastrointestinal and Liver Diseases and the USC Research Center for Liver Diseases, Keck School of Medicine, University of Southern California, Los Angeles, CA 90033, USA; ^4^Department of Cell Biology and Anatomy, College of Medicine, The University of Arizona, Tucson, AZ 85721, USA

## Abstract

Aging is associated with marked changes in the hepatic sinusoid, yet the effect of old age on hepatic stellate cells (HSC) has not been well described. Transmission electron microscopy and immunohistochemistry were used to study the effects of aging on HSC in livers from rats (3-4 mths versus 24–27 mths) and mice (2-3 mths versus 20–22 mths). Desmin-positive HSC doubled in old age in both mice and rats. Alpha-smooth muscle actin- (*α*SMA-) positive cells did not increase significantly and remained only a small percentage of desmin-positive cells. Electron microscopy revealed that old age is associated with HSC that have a substantial increase in the number of lipid droplets which are larger in diameter. There was also a marked increase of HSC that protruded into the sinusoidal lumen in old mice. In conclusion, old age is associated with hyperplasia of HSC that are not activated and are engorged with lipid droplets.

## 1. Introduction

Old age is associated with various changes in the hepatic sinusoid. These include thickening of the liver sinusoidal endothelial cell (LSEC) and loss of endothelial fenestrations, deposition of perisinusoidal basal lamina and collagen, and upregulation of proteins not usually seen in the young healthy liver such as von Willebrands factor and collagen [1, 2]. This has been termed age-related pseudocapillarisation. In addition, there are increased numbers of basally activated Kupffer cells (KC) that respond poorly to immune challenges [3]. As well as the LSEC and KC, the other main cell in the hepatic sinusoid is the hepatic stellate cells (HSC).

The HSC (or Ito cell) is a pericyte that resides in the extracellular space of Disse and has long cytoplasmic extensions that wrap around the LSECs. HSC contain many lipid droplets that are rich in vitamin A, which generates characteristic autofluorescence. In the early phases of many chronic liver diseases, HSC become activated and contribute to the fibrosis by producing extracellular matrix components such as collagen. In this activated state, HSC lose their lipid droplets and become myofibroblastic in appearance and stain positive for *α*-smooth muscle actin (*α*SMA) [4, 5]. It is unknown whether HSC contribute to the mild perisinusoidal fibrosis seen in old age. Furthermore, a recent study has shown that LSECs are involved in the regulation of activation of HSC [6] which raises the possibility that the age-related changes in the LSEC might impact on HSCs.

There have been only a few reports of the effects of old age on HSC. An early study found that HSC had more and larger lipid droplets in old rats [7], a finding that we also described more recently in nonhuman primates [8] and mice [9, 10]. Here, we investigated whether old age is associated with any change in the number or activation of HSC and quantified the changes in lipid droplets that accumulate in HSC in old age.

## 2. Methods

### 2.1. Animals

Rats were F344 aged 3–6 mths and 24–27 mths (*n* = 5 old, *n* = 6 young) obtained from the National Institute of Aging (Baltimore, USA). Mice were B10 aged 2–3 mths and 20–22 mths (*n* = 3 in each group) that were maintained at the Centenary Institute (Sydney, Australia) in specific pathogen-free conditions and fed standard laboratory chow. The study was approved by the Sydney Southwest Area Health Service Animal Welfare Committee. After ketamine/xylazine anesthesia, liver tissue was harvested and fixed with paraformaldehyde for immunohistochemistry or with glutaraldehyde for transmission electron microscopy.

### 2.2. Transmission Electron Microscopy

Two blocks of glutaraldehyde-fixed liver tissue from each animal were cut and studied using a Philips CM10 transmission electron microscope. Approximately 20 HSC for each animal were photographed at 2,600 magnification and subsequently analyzed using ImageJ (http://rsbweb.nih.gov/ij/). The larger diameter of each lipid droplet contained in the cell cytoplasm and the area of each cell was determined. The number of lipid droplets per HSC was also analysed only in those sections where the HSC contained a nucleus in an attempt to compensate for the possibility that the sections were taken in different planes. In total we analyzed 680 droplets and 108 HSC from young rats, 806 droplets and 98 HSC from old rats, 170 droplets and 59 HSC from young mice, and 351 droplets and 57 HSC from old mice.

### 2.3. Immunohistochemistry

Staining was performed for desmin, which is a marker of HSC, and *α*SMA, which is a marker of activated HSC [11]. Liver specimens embedded in paraffin blocks were used. Sections from each block were stained for desmin (NCL-DER11 Novocastra 1 : 200) and *α*SMA (NCL-SMA Novocastra 1 : 200). Rat liver sections were processed using an automatic IHC Leica staining protocol on Leica Bond Polymer, Refine Detection (DS9800) with Bond DAB enhancer 30 mL (AR9432). EDTA buffer pH 9 was used for the antigen retrieval step for 30 min for the detection of desmin. Mice sections were stained manually. For desmin visualization, slides were incubated for 10 min in microwave with the same antigen retrieval buffer (EDTA pH 9). Sections were pretreated with 0.3% hydrogen peroxide for 20 min in PBS and avidin-biotin blocking solutions and then incubated with primary antibodies over night at 4°C. Antimouse biotinylated secondary antibody (1 : 150 Sigma, St. Louis, MO) was applied for 40 min at room temperature followed by peroxidase-conjugated streptavidin (40 min, 1 : 50 Sigma, St. Louis, MO). Peroxidase activity was visualized using 3,3′-diaminibenzidine. Three representative images for each section were taken at 200 magnification and analyzed using the software ImageJ. For each photograph, parenchymal area was calculated (large vessels were excluded) and desmin- and *α*SMA-positive nucleated cells were manually counted, and results were adjusted for 0.5 mm^2^ area.

### 2.4. Statistics

The results are expressed as average ± standard deviation and comparisons performed using parametric and nonparametric, two-tailed Students *t*-test, depending upon the Schapiro Wilk test for normality (SigmaPlot v11).

## 3. Results

**Figure 1:**
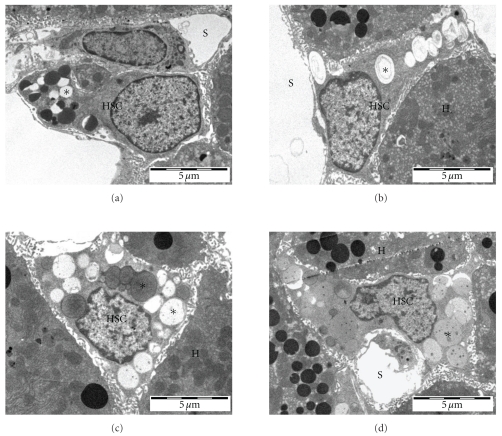
Representative transmission electron micrographs of HSC (HSC) from young (a,b) and old rats (c,d). Hepatic stellate cell, HSC; sinusoidal lumen, S; hepatocyte, H; lipid droplets*.

**Figure 2:**
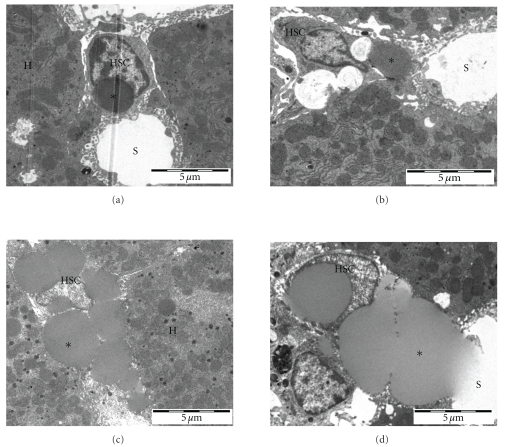
Representative transmission electron micrographs of HSC (HSC) from young (a,b) and old mice (c,d). Hepatic Stellate Cell HSC; Sinusoidal lumen S; Hepatocyte H; Lipid droplets*.

Table 1 shows the results of the transmission electron microscopic analysis and Figures 1 and 2 show representative pictures of stellate cells in young and old rats and mice. There were changes in HSC with old age in both rats and mice, although the changes were more dramatic in the mice. With old age, the number of lipid droplets per HSC increased, more so in mice. In addition, the average diameter of the lipid droplets increased in both the rats and mice, again more marked in the mice.

**Figure 3:**
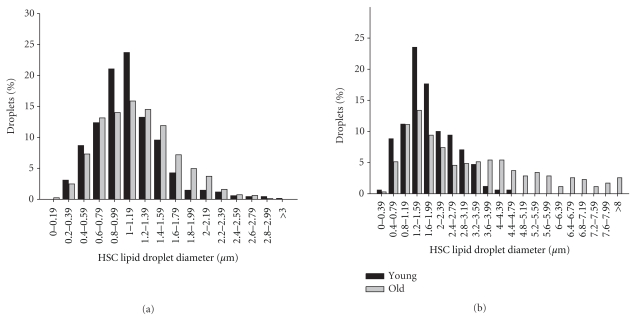
Frequency distribution of the diameter of the lipid droplets in HSC from rats (a) and mice (b). In both rats and mice, the distribution was skewed to the right in old age.

The frequency distribution of the diameter of the lipid droplets is shown in Figure 3. There appeared to be a normal distribution of the diameter of the lipid droplets in young animals, and the droplets were larger in mice than in rats. In old age, the distribution was skewed to the right with an appreciable number of much larger droplets.

Transmission electron microscopy also revealed that there was an increase in the percentage and number of enlarged HSC that resulted in protrusion of the endothelial lining into the sinusoidal lumen in old mice, but not in old rats.

**Figure 4:**
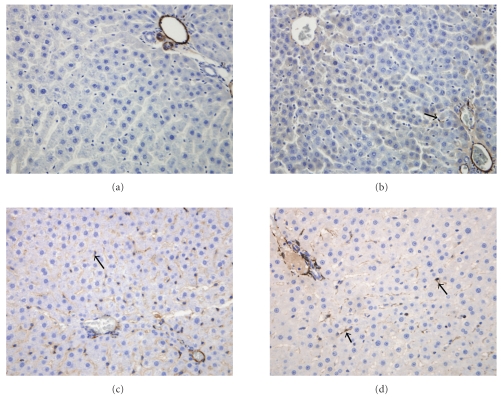
Representative immunohistochemistry from rat livers showing SMA in young (a) and old (b) rats, and desmin immunostaining in young (c) and old (d) rats (magnification ×200).

**Figure 5:**
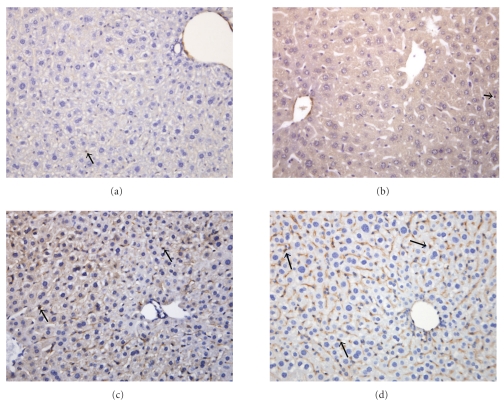
Representative immunohistochemistry from mice livers showing SMA in young (a) and old (b) mice, and desmin immunostaining in young (c) and old (d) mice (magnification ×200).

Table 2 shows the results of the immunohistochemistry analysis and Figures 4 and 5 show representative pictures of desmin and *α*SMA staining in livers from young and old rats and mice. HSC were more numerous in mice than rats. With old age, there was an approximate doubling of the numbers of HSC. Activated HSC as determined by *α*SMA represented only a very small percentage of total HSC. Although there was an increase in *α*SMA-positive cells with old age in both rats and mice, this was not statistically significant nor did it represent a substantive change in the fraction of HSC that is activated. There was a small zonal variation in *α*SMA staining which was more intense in zone 2 (mid zone), but age did not have any impact on this distribution.

## 4. Discussion

Old age is associated with an increase in the number of HSC and and in the size and number of the lipid droplets that they contain, but no change in their activation status.  Table 3 shows the results of all other studies that we could only find that make any mention of the effect of old age on HSC. There have been five other studies involving rats [7, 12], baboons [8], and mice [9, 10] that described changes in HSC lipid droplets in old age. In our study, we quantified these changes. In old age, there was an increase in the number of droplets per HSC by about 30% in rats and 250% in mice. The diameter of the droplets increased by about 10% in rats and 35% in mice. Lipid droplets in HSC contain about 80% of all retinoids in the body (vitamin A and its metabolites) mostly in the form of retinyl palmitate [4]. In addition, HSC lipid droplets contain triglyceride, cholesteryl ester, cholesterol, phospholipids, and free fatty acids. Retinyl ester and triglyceride are present at similar concentrations and together account for three-quarters of the total lipid in HSC lipid droplets [13]. In humans, plasma retinoids do not decrease with old age [14, 15], however, the postprandial levels are abnormal and consistent with reduced mobilization of vitamin A from the liver [15]. Vollmar et al. [16] reported an increase in retinoid content in old rat livers—a twofold increase of retinol, 32-fold increase of retinyl stearate, 53-fold increase of retinyl palmitate, and 66-fold increase of retinyl oleate. This suggests that the increase in the size and number of lipid droplets in HSC is related at least in part to an increase in retinoids in HSC. However, droplets also contain a similar amount of triglycerides, and there are reports of increased triglyceride content of old livers [17], but whether there is accumulation of triglycerides in HSC is not known.

There were increased HSC numbers in old age in both rats and mice. Previously, Enzan et al. [7] commented that there was no change in the frequency of HSC, but this study was performed using electron microscopy and no data were provided [7]. Similarly Martin et al. [18] concluded that there were no changes in HSC numbers in old age in rats. However, in their study, the volume of liver consisting of HSC increased from 0.43 ± 0.13% in young rats to 1.0 ± 0.24% in old rats but this did not reach statistical significance (*P* = .075 with only 4 rats in each age group) [18]. In our study, desmin immunohistochemistry was used to determine the number of HSC present in liver. In both rats and, mice old age was associated with approximately a doubling in the numbers of desmin-positive HSC per 0.5 mm^2^. As such, the result is numerically consistent with that of Martin et al., [18] although our results were statistically significant. It is of interest that we have previously found an increase in the number of Kupffer cells in old age. Specifically, the number increased from 2.0 ± 0.2 to 5.5 ± 0.6 cells per 29,500 *μ*m^2^ in old age in rats [3]. The effect of old age on the numbers of the other main cell of the hepatic sinusoid, the LSEC is unknown.

Finally, we performed immunohistochemistry with *α*SMA to determine whether these HSCs are activated. Activated HSCs are characterised by loss of lipid droplets and expression of *α*SMA. The fact that we and others have noted that HSC from old animals are engorged with lipid droplets is consistent with the conclusion that HSC are not activated in old age. Likewise, we did not find any statistically significant change in *α*SMA-positive HSCs in old age. Although there was a slight increase in the number of *α*SMA-positive cells in old age, this was balanced out by the doubling in the total number of HSC so that the fraction of total HSCs that is activated remained only very small. Previously Grizzi et al. [19] found a nonsignificant increase in *α*SMA in the livers of old rats, but this did not increase in response to carbon tetrachloride as was seen with young livers [19]. We also noted that *α*SMA staining was not increased in the livers of old baboons [8]. The lack of activation of HSCs in old age means that it is unlikely that these cells are contributing to the mild perisinusoidal fibrosis that is seen in many species in old age [2]. Furthermore, the fact that HSCs are quiescent in old age suggests that the other changes of pseudocapillarisation seen in the aged hepatic sinusoid are unlikely to be secondary to undiagnosed chronic liver disease because this is invariably associated with activation of HSCs.

In conclusion, old age is associated with increased numbers of fat engorged, quiescent HSCs. This has implications for the effect of age on retinoid metabolism and for the mechanisms for other age-related morphological changes in the hepatic sinusoid. The increase in the percentage and number of enlarged HSCs that resulted in protrusion of the endothelial lining into the sinusoidal lumen in old mice may contribute to the reduced sinusoidal blood flow reported in aged mice [10].

## Figures and Tables

**Table 1:** Transmission electron microscopic analyses of the effects of old age on HSC.

	Number of lipid droplets/HSC	Number of lipid droplets/HSC with nucleus	Diameter of lipid droplets in HSC (*μ*m^2^)	HSC protruding into sinusoidal lumen (number and % of total HSC)
Rats				
Young	6.76 ± 4.51	6.91 ± 4.59	1.08 ± 0.44	8 (7%)
Old	9.17 ± 6.35*	10.09 ± 6.38*	1.20 ± 0.50*	10 (10%)
Mice				
Young	2.41 ± 1.24	2.41 ± 1.24	1.83 ± 0.87	3 (5%)
Old	6.33 ± 3.55*	6.77 ± 4.31*	3.15 ± 2.19*	29 (51%)*

*P < .05.

**Table 2:** Results of immunohistochemical study of the effects of old age on HSC.

	Desmin staining (number of positive HSC/0.5 mm^2^)	*α*SMA staining (number of positive HSC/0.5 mm^2^)
Rats		
Young	21.3 ± 15.1	0.6 ± 1.0
Old	45.5 ± 23.5*	2.8 ± 3.8
Mice		
Young	66.1 ± 28.8	0 ± 0
Old	134.0 ± 55.6*	1.1 ± 1.8

*P < .05.

**Table 3:** The results of previous studies where the effect of old age on HSC was reported.

Citation	Species	Morphology	Number	Activation
[7]	Rat	↑ lipid droplets	↔	n.d.
[12]	Rat	↑ lipid droplets	n.d	n.d.
[18]	Rat	n.d.	↑ (from 0.43 to 1.02% of liver volume, *P* = .075)	n.d.
[16]	Rat	n.d.	↑ autofluorescence	n.d.
[8]	Baboon	↑ lipid droplets	n.d.	↔ (*α*SMA)
[19]	Rat	n.d.	n.d.	↔ (*α*SMA)
[9]	Mouse	↑ lipid droplets	n.d.	n.d.
[10]	Mouse	↑ lipid droplets	n.d.	n.d.

(n.d. not done).
